# Long-Term Experience with Acquired Haemophilia A: A 40-Year Single-Centre Study of Clinical Features and Outcome

**DOI:** 10.3390/jcm15010199

**Published:** 2025-12-26

**Authors:** Daniele Roselli, Giuseppe Malcangi, Maria Addolorata Bonifacio, Prudenza Ranieri, Renato Marino, Maria Addolorata Mariggiò

**Affiliations:** 1 Division of University Clinical Pathology, University Hospital Policlinico Consorziale, 70124 Bari, Italy; rosellidaniele94@gmail.com (D.R.);; 2 Haemophilia and Thrombosis Centre, University Hospital Policlinico Consorziale, 70124 Bari, Italy; 3 Section of Clinical Pathology, Department of Precision and Regenerative Medicine and Ionian Area, University of Bari Aldo Moro Medical School, 70124 Bari, Italy

**Keywords:** haemophilia A, factor VIII, inhibitor, prognosis, treatment, haemorrhage

## Abstract

**Background:** Acquired haemophilia A (AHA) is a rare autoimmune disorder characterized by the development of autoantibodies against Factor VIII activity, leading to a significant reduction in its functionality. Clinically, AHA presents with an unexpected prolongation of activated partial thromboplastin time (aPTT) and spontaneous bleeding episodes in patients without any personal or family history of haemorrhages. Bleeding manifestations can be severe at presentation, making early diagnosis and prompt treatment essential to reduce morbidity and mortality. **Methods:** We report on a single-centre cohort of 35 patients with AHA (examined from 1984 to 2024), analysing their demographics, underlying conditions, bleeding characteristics, treatment and outcome. **Results:** The median age of patients at diagnosis was 69 years (ranging from 18 to 92), 15 were males and 20 females. AHA was idiopathic in 37% of cases, severe bleeding was observed in 54% of patients treated with bypassing agents. Recombinant activated Factor VII (rFVIIa) was administered in 79% of cases and activated prothrombin complex concentrate (aPCC) in 10%, with no significant differences in haemostatic response and no thromboembolic complications. Occurrence of major bleeding showed no significant association with sex, age group, underlying condition, baseline Factor VIII activity or inhibitor titre at diagnosis. A total of 69% of patients were treated with corticosteroids alone, and 23% received a combination of corticosteroids and cyclophosphamide. Two patients died, six were lost to follow-up after partial remission, and one relapsed without bleeds after complete remission. Statistical analyses highlighted that the FVIII inhibitor titre > 20 BU was the only significant prognostic factor affecting time to complete remission. **Conclusions:** These observations emphasize the critical role of clinical suspicion and timely referral to experienced centres with adequate laboratory support for the effective management of AHA.

## 1. Introduction

Acquired Haemophilia A (AHA) is a rare autoimmune disease with an incidence of approximately 1.5 cases per million persons per year [1]. It is caused by the development of inhibitory autoantibodies against the plasmatic factor VIII activity (FVIII:C) leading to the typical haemorrhagic symptoms in patients without a family or personal history of bleeding [2]. The incidence of AHA typically exhibits a biphasic distribution, with one peak associated with pregnancy and a second occurring in individuals over 60 years of age. Approximately 50% of patients with AHA present with underlying conditions, most commonly other autoimmune diseases (rheumatoid arthritis, systemic lupus erythematosus and thyroid disorders) [3,4] or solid and lymphoproliferative malignancies (prostate, lung or colon cancers, leukemia, lymphoma and myeloma) [5,6,7,8].

In most cases, bleeding episodes are spontaneous, but they also occur after trauma or surgery. At diagnosis, bleeding is frequently severe and may pose a life-threatening risk, usually presenting as retroperitoneal or extensive intramuscular haematomas and in some cases, intracranial haemorrhages. In a minority of patients, bleeding manifestations are mild and do not require haemostatic intervention. The most commonly affected sites include soft tissues, muscle, skin and mucous membranes, whereas haemarthrosis is rarely observed [9].

Patients with AHA remain at risk of severe bleeding until inhibitory antibodies are completely eradicated [10]. For this reason, current treatment guidelines recommend the prompt initiation of immunosuppressive therapy (IST) using corticosteroids either as monotherapy or in combination with other immunosuppressive agents [11]. According to the most recent international guidelines on diagnosis and management of AHA, first-line IST should be individualized based not only on the patient’s performance status and comorbidities but also on prognostic markers known to influence treatment outcomes [2].

The multicentre study conducted in 2010 by the German, Austrian and Swiss Thrombosis and Haemostasis (GTH) Society reported that 80% of patients achieved partial remission (PR) within 21 days from the initiation of IST, particularly when baseline FVIII:C levels ≥ 1 IU/dL and inhibitor titres were <20 BU [12]. Other studies have identified several prognostic factors associated with the response to IST, including residual FVIII activity, high inhibitor titres, age > 65 years and underlying malignancy-associated conditions [13,14].

Here, we describe the clinical characteristics and management of a single-centre cohort of patients observed over a 40-year period, with the aim of identifying clinical and/or laboratory factors influencing treatment response and outcomes.

## 2. Materials and Methods

A single-centre retrospective study was conducted including 35 patients diagnosed with AHA and managed at our institution between 1984 and 2024. The diagnosis was established based on the presence of acute bleeding, unexplained isolated prolongation of activated partial thromboplastin time (aPTT) and normal prothrombin time, reduced FVIII activity (FVIII:C) and normal intrinsic coagulation factors assays.

The detection of FVIII inhibitor was confirmed using the Bethesda assay which measures the residual FVIII activity following incubation of equal volume of patient and normal pool plasma (NPP) at 37 °C for 120 min. A control mixture was prepared by incubating an equal volume of the NPP with imidazole buffer. The cut-off for positivity was set at 0.5 BU/mL [15]. All coagulation tests were performed on the ACL TOP500 CTS automated coagulation analyser (Instrumentation Laboratory, Milano, Italy).

The AHA diagnosis required plasma FVIII activity < 50% and the detection of the FVIII inhibitor, whose titre was determined using the one-stage FVIII assay and the Bethesda method [16].

Data recorded on each patient include demographics, underlying disorders, clinical course of diagnosis and management, clinical characteristics and treatment of haemorrhages, inhibitor eradication regimen and adverse events related to disease or treatment.

Major bleeding was defined as fatal bleeding or symptomatic bleeding in a critical area or organ, such as intracranial, intraspinal, intraocular, retroperitoneal, intra-articular, pericardial, intramuscular with compartment syndrome or a transfusion requirement of two or more units of whole blood or red blood cells [17]. The response to anti-haemorrhagic therapy was evaluated based on haemoglobin stabilization or increase, resolution of bleeding as assessed by physical examination and/or appropriate imaging techniques, the absence of therapy escalation and successful inhibitor eradication.

Complete Remission (CR) was defined as FVIII activity level > 50%, undetectable inhibitor titre, absence of bleeding and discontinuation of immunosuppressive therapy. Partial Remission (PR) was defined as FVIII activity > 50%, persistence of inhibitor without active bleeding and discontinuation of any haemostatic treatment for more than 24 h [12].

Statistical analyses were performed using R software, version 4.0.1. (R Development Core Team, Vienna, Austria) [18]. A *p*-value of less than 0.05 was considered statistically significant. The continuous quantitative variables were described using the median and interquartile range (IQR) and the qualitative variables were described using absolute and relative frequencies expressed as percentages. The Mann–Whitney U test was used to highlight median (IQR) differences in patient subgroups. Furthermore, to shed light on the variables impacting time to remission, the dataset was studied using multivariate Cox regression with scaled Schoenfeld residuals.

## 3. Results

### 3.1. Demographics

**Figure 1:**
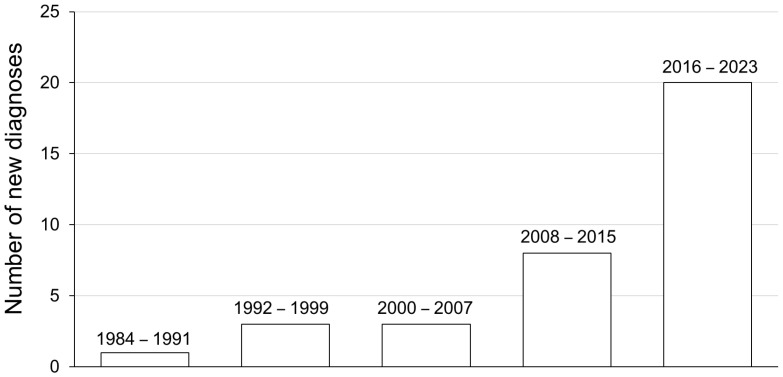
Incidence of newly diagnosed AHA cases over consecutive 7-year interval between 1984 and 2023.

A total of 35 patients were analysed between 1984 and 2024 at the Haemophilia and Thrombosis Centre of Bari University Hospital, referring to the Section of Experimental and Clinical Pathology of the same Hospital for laboratory assessment. A progressive increase in the incidence of diagnosed cases was observed over the years, starting from 2.8% (1/35) between 1984 and 1991 up to 57% (20/35) between 2016 and 2023 (Figure 1).

**Figure 2:**
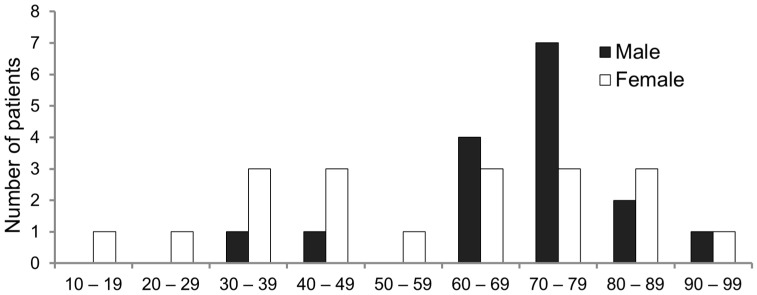
Distribution of patients with AHA according to age and sex.

The median age of patients at diagnosis was 69 (ranging from 18 to 92). Age and gender distribution demonstrated a low number of children and teenagers between our patients, with 69% of the total cohort aged over 60 years (Figure 2). A female preponderance was observed in this cohort (57%) and in the childbearing age group. However, a male preponderance was observed in the older age group (>60 years).

### 3.2. Associated Conditions

The concomitant diseases that could be associated with AHA are detailed in Table 1. The 37% of the patients (13/35) was regarded as idiopathic AHA without underlying disorders identified; solid malignancy and onco-hematologic diseases were reported in 8 patients (23%), 7 patients (20%) had autoimmune disorders, and 4 patients (11%) were in the postpartum period.

### 3.3. Laboratory Findings

**Figure 3:**
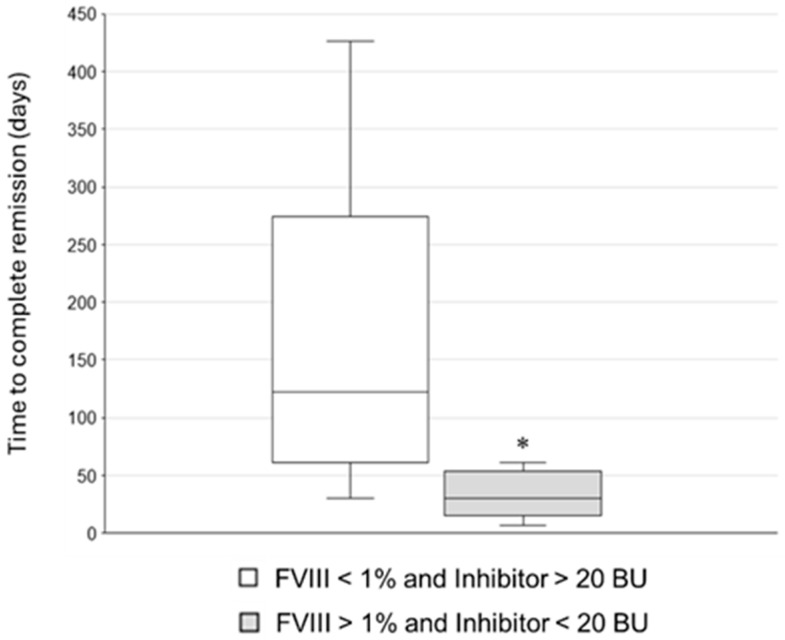
Box and whisker plot representing time to complete remission in two patient subgroups, as indicated in the legend. The Mann–Whitney U test with continuity correction was used to demonstrate a statistically significant difference (U = 106, * *p* = 0.007) among the median time to CR of the two subgroups (122 vs. 30 days).

FVIII activity at diagnosis was undetectable in 21 patients (60%) (Table 2). The inhibitor level range at diagnosis was 1–2100 BU (median 20 BU, IQR 4–95 BU) while FVIII:C was between <1% and 12%. A difference of time to reach CR was observed in patients with FVIII < 1% and inhibitor titres > 20 BU and patients with FVIII > 1% and Inhibitor titres < 20 BU (*p* = 0.007). Indeed, the latter achieved CR in a median time of one month, while high inhibitor titres and low levels of FVIII activity were associated to a median time to CR equal to four months (Figure 3).

### 3.4. Bleeding Characteristics

At diagnosis, all patients exhibited active bleeding. Spontaneous subcutaneous haemorrhages involving multiple (≥2) anatomical sites were commonly observed, whereas haemarthrosis was not reported (Table 3).

More than half patients (54%) were treated with haemostatic therapy. Major bleeding was not associated with sex, underlying disease and age group. No statistically significant differences were observed in the mean inhibitor titre at diagnosis or in factor VIII concentration between patients who experienced major bleeding events and those who did not (*p* > 0.05) (Table 4).

### 3.5. Haemostatic Therapy

Nineteen patients received haemostatic therapy for major bleeding: 15/19 (79%) underwent treatment with recombinant activated Factor VII (rFVIIa) and 2/19 (10%) with activated prothrombin complex concentrate (aPCC). The response rate was 87% and 100%, respectively (Table 5). One patient who did not respond to rFVIIa was successfully switched to recombinant porcine FVIII (rpFVIII). No statistically significant differences in response rate were observed among the different therapies.

### 3.6. Immunosuppressive Therapy

**Figure 4:**
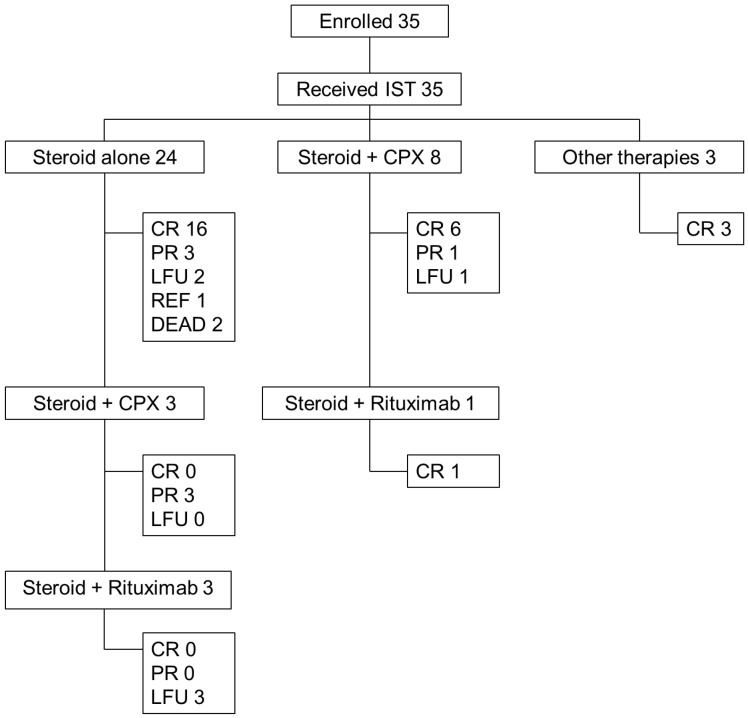
Flow chart and outcome of immunosuppressive therapies. Abbreviations: IST, immunosuppressive therapy; CPX, cyclophosphamide; CR, complete remission; PR, partial remission; LFU, lost to follow up; REF, refractory.

All 35 patients were treated with immunosuppressive therapy (IST) to eradicate inhibition. Two patients died after a short treatment with corticosteroids, one had refractory status without bleeds. The treatment flowchart of patients is shown in Figure 4.

Twenty-four patients were treated with steroids alone, with a 66% response rate, and eight patients were treated with both steroids and cyclophosphamide, with a 75% response rate (Table 6).

Three patients who exhibited inadequate response to corticosteroid monotherapy subsequently received combination therapy with corticosteroids and cyclophosphamide, followed by rituximab as third-line IST. These patients demonstrated only partial clinical responses and were subsequently lost to follow-up. In contrast, one patient initially treated with corticosteroids and cyclophosphamide achieved complete remission following a therapeutic switch to rituximab. Between 1984 and 1995, three additional patients were managed with corticosteroids, intravenous immunoglobulins and plasma-derived porcine FVIII (pFVIII); all of them achieved complete remission after a treatment mean duration of 18 months. One patient developed neutropenia as an adverse event following corticosteroid and cyclophosphamide therapy. Treatment was subsequently modified to corticosteroid monotherapy, but the patient was lost to follow-up after attaining partial remission.

### 3.7. Prognostic Factors for Remission

**Figure 5:**
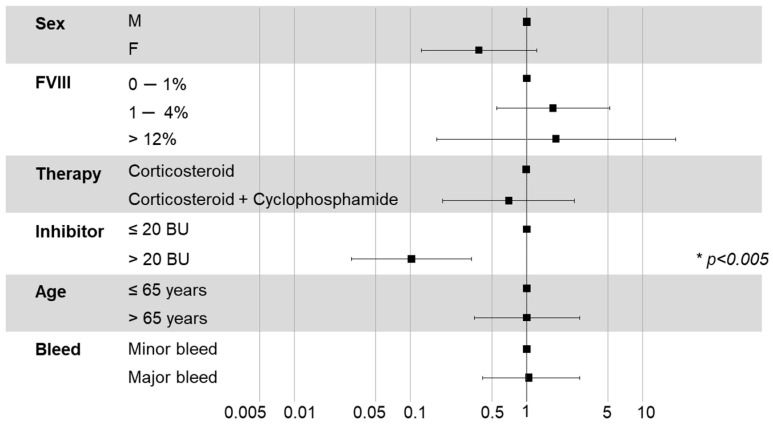
Forest plot showing the impact of each variable on complete remission (multivariate Cox analysis). The asterisk refers to a statistically significant difference (*p* < 0.005).

**Figure 6:**
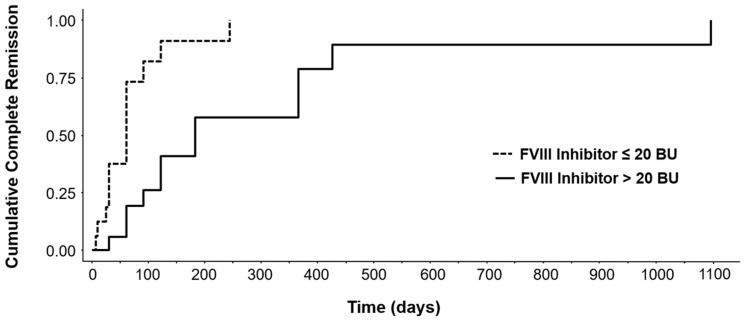
Cumulative time of complete remission in patients with inhibitor titres < 20 BU (dotted line) and patients with inhibitor titres > 20 BU (solid line).

Overall, 26 patients achieved CR and the median time to CR was 60 (10–1080) days. Multivariate Cox regression analysis for time to CR showed that FVIII inhibitor > 20 BU was the only significant prognostic factor, as indicated in the forest plot (Figure 5). A patient with low inhibitor titre was likely to achieve CR ten-times before a patient with high inhibitor titre (HR = 0.102, *p* < 0.001, CI = 0.84, log-rank global *p* = 0.002). Other factors, including major bleeding and FVIII level, did not affect time to CR. Patients with FVIII > 12% showed a broader variability of time to CR. Observing the cumulative occurrence of complete remission, the difference between the two subgroups of patients (inhibitor titres over and below 20 BU) is highlighted (Figure 6). Moreover, patients with inhibitor titres > 20 BU took longer to achieve haemostasis than those with FVIII inhibitor titres ≤ 20 BU (median of 10 days vs. 7 days, respectively). Notably, no significant differences in reaching CR were observed between patients in treatment with corticosteroids vs. corticosteroids plus cyclophosphamide.

## 4. Discussion

This study presents the experience of a single centre cohort, including 35 patients treated in 40 years, which support the rarity of condition. Diagnoses increased between 2016–2023, likely because of lack of diagnostic suspicion by clinicians in previous years, resulting in patients sent late to our centre from district hospitals, especially those without specialised laboratory facilities able to assess FVIII and its inhibitor titre. Furthermore, the use of anticoagulant treatment could also complicate AHA diagnosis [19]. Data present a similar overall AHA distribution in men and woman. However, AHA was more frequent in male patients over 60 years, with a second peak being observed in female patients aged 20–40 years, corresponding to postpartum [20]. Our dataset is consistent with the findings described by other authors referring to larger cohorts, i.e., the low incidence of AHA in children, the unknown aetiology for one third of the patients and the frequent association with malignancies in elderly patients [3,9,12,21].

However, these studies observed that autoimmune diseases were the most common condition in young patients. Conversely, pregnancy was the most common underlying condition in our group of young patients. Indeed, in this study, patients with autoimmune diseases have a median age of 76.

All of our patients presented at least one bleeding event, with subcutaneous bleeding being the most common bleeding site, as confirmed in larger cohorts [22,23]. Rare bleeding was observed in the gastrointestinal tract, genitourinary tract and oral mucosa. No intracranial haemorrhage or joint bleeding were observed. Therefore, bleeding patterns differed from that of patients with congenital haemophilia A [24]. Spontaneous bleeding occurred in a high percentage of cases (80%). A total of 54% of patients had major bleeding, not associated with other variables, in agreement with the findings from the UKHCDO and EACH2 studies [3,9].

No statistically significant differences in the response rate to different haemostatic therapies (rFVIIa vs. aPCC) were noted, as already reported by Kessler and Knöbl [25]. One patient not responding with rFVII was switched with success to recombinant porcine factor VIII (rpFVIII); she was 53 years old with a superficial thrombosis in the past with a large haematoma on the upper arm and iliopsoas muscle and a *K. pneumonia* sepsis [26]. Administration of rpFVIII avoided the risk of compartment syndrome and it was a useful addition to the limited treatment options available for the management of acute bleeding episodes in adults with AHA [27]. Since this is a retrospective study spanning from 1984 to 2023, emicizumab was not used in our patients, but it represents a recent therapeutic option for prophylaxis of bleeding recurrences [28].

Excluding the patients lost to follow-up, in this study the overall mortality rate was 5.7% (*n* = 2). These rates are similar to previous registry data (4–11%, infection; 3.5–9.1%, bleeding) [3,9,12].

The two deaths occurred after a short time from diagnosis. An 80-year-old male with hypertension, diabetes, hypercholesterolemia and diverticulosis in anamnesis developed an enterorrhage and a haematoma of the iliopsoas muscle, acute renal failure and a pleural effusion that ended with a hypovolemic and septic shock without any chance of survival, despite haemostatic therapy. A second male patient, 74 years old, had an aortic valve prosthesis, a descending aortic prosthesis with multiple haematomas. He died due to a sudden heart attack after inhibitor eradication during IST tapering. Both dead patients were treated with IST having an inhibitor at the diagnosis of 35 BU and 30 BU, respectively. Another male patient, 78 years old, with a previous heart attack had a relapse AHA without symptoms but with altered laboratory analyses after seven months of complete remission. Corticosteroids for two months eradicated inhibitor of 2 BU. Tiede et al. [12] underlined that infections were the foremost cause of death for AHA patients. However, our cohort could not confirm these data, due to the reported low cases of deaths.

More than half the patients with inhibitor ≤ 20 BU and >20 BU were treated as first line patients, with corticosteroids and corticosteroids + cyclophosphamide, respectively [2]. A patient who was treated first with steroids and cyclophosphamide needed the administration of rituximab to achieve complete remission. In the first multicentre randomized controlled trial, a single dose rituximab plus glucocorticoid regimen showed similar efficacy and safety, without a reported risk of secondary malignancies or reproductive toxicity seen in patients treated with cyclophosphamide, thus it might be recommended as a first-line therapy for AHA [29]. From 1984 to 1995, three patients with a mean inhibitor titre of 186 BU achieved CR after a mean of 18 months, requiring the administration of corticosteroid, intravenous immunoglobulins and purified pFVIII concentrate, a therapeutic agent conventionally employed in haemophilia A patients with inhibitors [30]. Currently, recombinant porcine FVIII (rpFVIII) has a purity greater than 99%, which significantly reduces the risk of adverse events, such as thrombocytopenia and hypersensitivity reactions, commonly associated with purified porcine FVIII concentrate. Moreover, rpFVIII demonstrates markedly lower cross-reactivity with human anti-FVIII inhibitors [30,31,32], making it a safer and more targeted therapeutic option in patients with AHA [33].

We showed that the FVIII inhibitory antibody titre was a predictor of achieving early CR after IST. Patients with >20 BU of FVIII inhibitors needed ten times longer to achieve complete haemostasis and CR. Other studies report that FVIII inhibitory antibody titres > 20 BU/mL are a predictor for the response to IST [12,14]. Hyun et al. suggested that patients with high inhibitor titres are less likely to achieve CR with steroid monotherapy [34]. In our cohort, no significant differences were observed between patients in treatment with corticosteroids versus corticosteroids plus cyclophosphamide in reaching CR, but there were 3 patients with ≤20 BU treated with corticosteroids alone and 2 patients with >20 BU treated with corticosteroids plus cyclophosphamide.

The CR rate after IST was 74%, which was similar to that reported in previous studies [3,9,12,21]. Factors such as FVIII levels < 1 IU/dL or high inhibitor titres have been proposed to be the predictors for poor response to IST [35]. However, there is a discrepancy among studies regarding the criteria to define a high inhibitor titre. Inhibitor titres < 16 BU/mL and higher FVIII levels were reported as predictors of faster response in the EACH2 registry data [3]. Moreover, in the prospective GTH 2010 study, inhibitor titres ≥ 20 BU/mL were reported as a poor predictor of CR. However, in patients with FVIII levels of ≥1 IU/dL and inhibitor titres < 20 BU/mL, the probability of achieving PR within 21 days with steroid monotherapy was 11 times higher [12]. A French study also reported that the time to achieve PR was shorter (15 days vs. 41 days) in patients with FVIII levels ≥1 IU/dL and inhibitor titres < 20 BU/mL after IST with steroid alone [14]. In agreement with the GTH 2010 study, the present study revealed inhibitor titres > 20 BU/mL as a predictor of response to IST. Our data are consistent with the 2020 international recommendations on AHA that suggest inhibitor titres ≥ 20 BU/mL as one predictor of poor response to IST [2]. On the other hand, the risk of bleeding cannot be predicted by baseline FVIII level or baseline inhibitor titres. However, the recovery of the weekly evaluated FVIII level was reported to predict the risk of recurrent or subsequent bleeding [24]. In this respect, achieving PR (FVIII ≥ 50%) significantly reduced the risk of bleeding [36].

Our study has several limitations, primarily related to the small cohort size (*n* = 35). This limitation is intrinsically linked to the low incidence of AHA and the single-centre design of the investigation. Furthermore, none of the included patients received treatment with emicizumab, the bispecific monoclonal antibody which was introduced into clinical practice in late 2017 [37]. Similarly, only one patient was treated with susoctocog α. Future research will likely focus on emicizumab and new monoclonal therapeutic agents, discussing their impact on the evolving standard of care for individuals with AHA.

## 5. Conclusions

The presented results confirm the high response rate and the high rate of 12-month overall survival reported in the main registries. Moreover, patients with FVIII inhibitory antibody levels > 20 UB took longer to achieve haemostasis and complete remission.

## Figures and Tables

**Table 1:** Patient characteristics at the time of diagnosis.

Characteristics	Patients (*n* = 35)
Age median (range)	69 (18–92)
Gender (Male/Female)	15/20 (43/57%)
Idiopathic disorder	13 (37%)
Pregnancy	4 (11%)
***Autoimmune diseases***	7 (20%)
Systemic lupus erythematosus	2 (6%)
Rheumatoid arthritis	1 (3%)
Polymyalgia rheumatica	2 (6%)
Autoimmune hepatitis	2 (6%)
***Onco-hematologic diseases***	5 (14%)
Myeloma	2 (6%)
Chronic lymphoid leukaemia	2 (6%)
Chronic myeloproliferative syndrome	1 (3%)
***Cancer***	3 (9%)
Lung cancer	2 (6%)
Renal cancer	1 (3%)
Cured malignancy	1 (3%)
***Infections***	3 (9%)
Chronic hepatitis C	2 (6%)
Osteomyelitis	1 (3%)
***Comorbidities***	
Atrioventricular block	1 (3%)
Hypertension	9 (25.7%)
Heart attack	3 (8.6%)
Atrial Fibrillation	3 (8.6%)
Mechanical aortic prosthesis	2 (6%)
Aorta prosthesis	1 (3%)
Renal failure	5 (14.3%)
Benign prostatic hyperplasia	2 (6%)
Epilepsy	1 (3%)
Parkinson’s disease	2 (6%)
Medically assisted procreation	1 (3%)
Polycystic ovary syndrome	1 (3%)
Endometriosis	1 (3%)
Osteoporosis	1 (3%)
Type 2 diabetes mellitus	10 (28%)
Chronic obstructive pulmonary disease	2 (6%)
Obesity	1 (3%)
Pulmonary fibrosis	1 (3%)
Iron deficiency	1 (3%)
Thalassemia	2 (6%)
Deep venous thrombosis	1 (3%)
Superficial venous thrombosis	2 (6%)

**Table 2:** Distribution of FVIII levels and FVIII inhibitor titres of AHA patients.

FVIII:C (%)	Frequency
<1	21 (60%)
1–5	12 (34%)
>5	2 (6%)
**FVIII inhibitor titre (1–2100 BU)**	**Frequency**
≤20	18 (51%)
>20	17 (48%)

**Table 3:** Characteristics associated with bleeding symptoms.

Symptoms	Patient *n.* (%)
**Presence of bleeding symptoms**	35/35 (100)
*Severity of bleeding* ^a^	
Major	19/35 (54)
Minor	16/35 (46)
*Number of bleeding sites*	
1	12/35 (34)
≥2	23/35 (66)
*Cause of bleeding*	
Spontaneous	28/35 (80)
Surgery	3/35 (9)
Puerperium	4/35 (11)
*Bleeding site*	
Subcutaneous	24/35 (69)
Muscle	11/35 (31)
Gastrointestinal tract	1/35 (3)
Genitourinary tract	3/35 (9)
Mucosa	1/35 (3)

^a^ Major bleeding: fatal or symptomatic bleeding in a critical area or organ (see the Materials and Methods section).

**Table 4:** Bleeding association.

Underlying Condition	Mayor Bleeding *n*. (%)	Minor Bleeding *n*. (%)
Idiopathic disorder	9 (26%)	4 (11%)
Pregnancy	1 (3%)	3 (9%)
Autoimmune disease	2 (6%)	5 (14%)
Onco-hematologic disease	3 (9%)	2 (6%)
Cancer	2 (6%)	1 (6%)
Infection	2 (6%)	1 (6%)
*Age (years)*		
<65	6 (17%)	9 (26%)
≥65	10 (28%)	10 (28%)
*Sex*		
Male	8 (23%)	7 (20%)
Female	8 (23%)	12 (34%)
*Bleeding sites*		
1	7 (20%)	5 (14%)
≥2	10 (28%)	13 (37%)
FVIII:C (%)		
<1	12 (34%)	9 (26%)
1–5	5 (14%)	7 (20%)
*FVIII inhibitor titre (BU)*		
≤20	6 (17%)	12 (34%)
>20	11 (31%)	6 (17%)

**Table 5:** Summary of haemostatic treatment.

	Overall	Haemostatic Efficacy
**First line treatment**	(*n* = 19/35) 54%	
*Bypassing agents*		
rFVIIa	15/19 (79%)	87%
aPCC	2/19 (10%)	100%
*FVIII concentrates*		
pFVIII	2/19 (10%)	100%
*Second line treatment*		
pFVIII	1/19 (5%)	100%
rpFVIII	1/19 (5%)	100%

rFVIIa: recombinant activated factor VII; aPCC: activated prothrombin complex concentrate; pFVIII: plasma-derived porcine factor VIII; rpFVIII: recombinant porcine FVIII.

**Table 6:** Complete remission rates of first line immunosuppressive therapies.

**Regimen**	**Treated Patients**	**Inhibitor Titre ≤ 20 BU (*n*/Treated)**	**Inhibitor Titre > 20 BU (*n*/Treated)**
Patients		18/35	17/35
Steroid	16/24	13/16	3/16
Steroid + CPX	6/8	2/8	4/8
Steroid + Rituximab	0	0	0

CPX = cyclophosphamide; BU = Bethesda Unit.

## Data Availability

Raw data are available from the corresponding author, on reasonable request.
